# Draft Genome Sequence of *Streptococcus dysgalactiae* subsp. *equisimilis* Strain C161L1 Isolated in Vellore, India

**DOI:** 10.1128/genomeA.00336-17

**Published:** 2017-05-11

**Authors:** Anshu Babbar, D. Patric Nitsche-Schmitz, Dietmar H. Pieper, Israel Barrantes

**Affiliations:** Microbial Interactions and Processes Research Group, Helmholtz Centre for Infection Research, Braunschweig, Germany

## Abstract

*Streptococcus dysgalactiae* subsp. *equisimilis* belongs to the β-hemolytic group C and G pyogenic group of streptococci. Here, we report the draft genome of the *S. dysgalactiae* subsp. *equisimilis* strain C161L1 from Vellore, a region in southern India with a high incidence rate of *S. dysgalactiae* subsp. *equisimilis* infection. This genome is 2.1 Mb long, with a 39.82% G+C content, and encodes 2,022 genes.

## GENOME ANNOUNCEMENT

*Streptococcus dysgalactiae* subsp. *equisimilis* is an emerging human pathogen that causes clinical manifestations similar to infections caused by *S. pyogenes* and in some cases has also been implicated in severe invasive infections such as necrotizing fasciitis and streptococcal toxic shock syndrome (1). Here, we present the draft genome of *S. dysgalactiae* subsp. *equisimilis* strain C161L1, which was isolated from throat samples in 2006 from Vellore, a region in southern India that is known to have a relatively high incidence rate of *S. dysgalactiae* subsp. *equisimilis* infections (2).

The genome was sequenced by Illumina MiSeq paired-end sequencing (250-bp per pair), producing 2,325,040 reads and entailing 583,585,040 bp for each sequencing pair. Adapters and other control fragments were removed from the sequencing data after the initial quality check (3). Later, quality trimming was performed using default values for paired-end sequencing (4–7). Subsequently, the trimmed sequencings were assembled using multiple *k*-mer sets (8). This resulted in 319 contigs, which were then annotated (9). During the annotation, two contigs were filtered out for compliance with the public databases. The final assembly comprises 2,121,729 bp and contains no unambiguous bases; contigs over 0.5 kb possess a G+C content of 39.82% and an *N*_50_ of 61,959 bp (10). These values for genome size and G+C content are in agreement with those of the five complete *S. dysgalactiae* subsp. *equisimilis* genomes in GenBank, which, on average, are 2.13 Mb in size and have a 39.49 to 39.5% G+C content (11). The annotation was carried out using only proteins encoded in existing reference genomes for this species as targets. Here, we found 2,022 genes, distributed among protein-coding genes (*n* = 1,972), tRNAs (*n* = 47), a single tmRNA molecule, and 2 ribosomal RNAs. These values are also within the expected range (11).

As of now, C161L1 is the only representative sequenced from Vellore, India. Therefore, this draft genome will provide insight into the genetic composition and structure of *S. dysgalactiae* subsp. *equisimilis* strains from Vellore, India, and will be useful for comparing and contrasting strains from various geographic locations, especially Europe, where the incidence rates are lower.

### Accession number(s).

Sequencing reads, assembled contigs, and annotations were deposited in the EMBL-EBI European Nucleotide Archive (ENA) under the accession numbers FWEH01000001 to FWEH01000317.
